# ASO Author Reflections: Indocyanine Green Fluorescence Navigation in Liver Surgery: Current Applications and Future Perspectives

**DOI:** 10.1245/s10434-022-13066-y

**Published:** 2023-01-13

**Authors:** Fusheng Liu, Yufeng Yuan

**Affiliations:** 1 Department of Hepatobiliary and Pancreatic Surgery, Zhongnan Hospital of Wuhan University, Wuhan, Hubei People’s Republic of China; 2 Clinical Medicine Research Center for Minimally Invasive Procedure of Hepatobiliary & Pancreatic Diseases of Hubei Province, Wuhan, Hubei People’s Republic of China; 3 TaiKang Center for Life and Medical Sciences, Wuhan University, Wuhan, Hubei People’s Republic of China

## Past

In the last decade, the concept of precision surgery has led to the development of hepatectomy guided by visual and quantifiable means. Both the liver reserve function and residual volume are quantitatively assessed before surgery using Child-Pugh grading, the indocyanine green (ICG) retention rate, and 3D reconstruction techniques. In addition, ICG fluorescence navigation, intraoperative ultrasound, and virtual reality technology are used for intraoperative visualization. As a widely used visualization technique, ICG fluorescence was introduced in 2010 for the visualization of liver tumors during laparoscopic hepatectomy.^1^ It has subsequently been used for segmental or lobar visualization of the liver. ICG fluorescence navigation has been technically refined over the past decade, including the dose, method, and timing of ICG administration, as well as the ICG fluorescence navigation-based protocols for laparoscopic liver resection.^2,3^ However, there is still a lack of evidence on whether liver resection guided by ICG fluorescence navigation improves the long-term prognosis of patients with liver cancer.

## Present

As far as the technical level is concerned, the use of ICG fluorescence navigation for guiding hepatectomy has been gradually standardized and it is clear that it improves the both the efficiency and safety of hepatectomy. In recent years, the technique has also been widely used in hepatectomy involving difficult sites, such as right posterior lobectomy, S7 segmentectomy, and S8 segmentectomy.^2^ However, while the technical advantages of the technique are apparent, there is limited information on the long-term benefits of the technique for patients with liver cancer. Our recent research suggested that ICG fluorescence-guided liver resection improves recurrence-free survival and may contribute to improved long-term prognosis.^4^ The reasons for the beneficial effects of ICG fluorescence navigation on patient survival and prognosis may include: (1) the ability of intraoperative fluorescence to detect lesions that are not apparent on preoperative imaging; (2) improvement in the rate of radical resection; and (3) guidance of the exact plane of hepatic resection to obtain wider margins.

## Future

Although ICG fluorescence navigation has proved to have significant value, the current fluorescence navigation techniques have several disadvantages that could be improved. These include the weak penetration of near-infrared-I region fluorescence (NIR-I, 700–900 nm), limited detection depth of imaging, and low resolution of imaging. The development of novel, biocompatible chemiluminescent substances that emit near-infrared-II region fluorescence (NIR-II, 1000–1700 nm) is required.^5^ Since the currently used ICG fluorescence navigation technology does not have the ability to target hepatocellular carcinoma, especially in patients with cirrhosis where there is a high false-positive detection rate, there is a need to develop fluorescent probes with tumor specificity to screen for tumor-specific targets. These new fluorescent probes should be tumor-specific and able to overcome the limitations of ICG and other imaging agents. The corresponding directions in basic research include the screening of tumor cells themselves, identification of tumor-related genes, and investigation of the tumor microenvironment.

## References

[1] Ishizawa T, Bandai Y, Harada N, Muraoka A, Ijichi M, Kusaka K, Shibasaki M, Kokudo N (2010). Indocyanine green-fluorescent imaging of hepatocellular carcinoma during laparoscopic hepatectomy: an initial experience. Asian J Endosc Surg.

[2] Li J, Li X, Zhang X (2022). Indocyanine green fluorescence imaging-guided laparoscopic right posterior hepatectomy. Surg Endosc.

[3] Felli E, Ishizawa T, Cherkaoui Z (2021). Laparoscopic anatomical liver resection for malignancies using positive or negative staining technique with intraoperative indocyanine green-fluorescence imaging. HPB (Oxford).

[4] Liu F, Wang H, Ma W, et al. Short- and long-term outcomes of indocyanine green fluorescence navigation- versus conventional-laparoscopic hepatectomy for hepatocellular carcinoma: a propensity score-matched retrospective cohort study. *Ann Surg Oncol.* 2023. 10.1245/s10434-022-13027-5. 10.1245/s10434-022-13027-5 PMC10027802 36645540

[5] Yang R-Q, Lou K-L, Wang P-Y (2021). Surgical navigation for malignancies guided by near-infrared-II fluorescence imaging. Small Methods.

